# Techno-Economic Feasibility of Functional Snacks from Brewer’s Spent Grain and Sweet Potato: A Simulation Study

**DOI:** 10.3390/foods15101654

**Published:** 2026-05-09

**Authors:** Alberto Ordaz, Analaura Gómez-Cisneros, Anayansi Escalante-Aburto, Mariel Calderón-Oliver

**Affiliations:** 1 Tecnologico de Monterrey, Escuela de Ingeniería y Ciencias, Campus Estado de México, Av. Eugenio Garza Sada 2501, Monterrey 64849, Nuevo León, Mexico; alberto.ordaz@tec.mx; 2 Tecnologico de Monterrey, Escuela de Ingeniería y Ciencias, Campus Toluca, Av. Eugenio Garza Sada 2501, Monterrey 64849, Nuevo León, Mexico; a01364924@tec.mx (A.G.-C.); anayansi.escalante@tec.mx (A.E.-A.); 3 Tecnologico de Monterrey, Institute for Obesity Research, Campus Monterrey, Av. Eugenio Garza Sada 2501, Monterrey 64849, Nuevo León, Mexico

**Keywords:** process simulation, SuperPro designer, *Ipomoea batatas*, cereals, sensitivity analysis, food industry

## Abstract

This study evaluates the techno-economic feasibility of producing a functional baked snack formulated with sweet potato flour, cereals, and upcycled brewer’s spent grain (BSG). The analysis, developed in SuperPro Designer^®^, integrates experimentally derived parameters from literature, justifying the transition from laboratory-scale data to an industrial production model. The analysis identified refrigerated storage (48 h) and tray drying as the primary bottlenecks limiting throughput. By synchronizing equipment cycles and increasing the number of units, the production capacity was adjusted from 154.32 to 1077.21 metric tons per year, capturing approximately 0.8% of the estimated annual demand for sweet potato snacks in Mexico. Economic evaluation for this scale demonstrated a capital investment of USD 24.6 million and annual operating costs of USD 8.49 million. The inclusion of a sedimentation-based water treatment, while increasing costs, enables a significant reduction in freshwater intake. The project yielded a payback period of 3.62 years and a Net Present Value (*NPV*) of USD 23.908 million. Sensitivity analysis revealed that profitability is strongly influenced by production volume and sweet potato costs. These findings provide a realistic framework for assessing the commercial viability of functional food formulations when scaled for industrial production.

## 1. Introduction

The global food industry faces a growing challenge in balancing consumer demand for convenience foods with the need for improved nutritional quality and sustainable production systems. Conventional snack products are often characterized by high levels of refined carbohydrates and fats, and low dietary fiber, which are associated with an increased risk of chronic diseases [1]. In response, current trends in food innovation are shifting toward developing functional snacks that incorporate nutrient-dense ingredients and promote environmentally responsible production practices.

Among the strategies to achieve this goal, the valorization of agro-industrial by-products has gained increasing attention within the framework of circular economy approaches. Brewer’s spent grain (BSG), the primary by-product of the brewing industry, represents approximately 85% of total residues generated during beer production [2]. BSG is rich in dietary fiber, protein, and bioactive compounds, making it a promising ingredient for enhancing the nutritional profile of food products [2,3]. Several studies have highlighted its potential to contribute to daily fiber intake and improve functional properties in food formulations [2,3,4].

Despite its nutritional advantages, the incorporation of BSG into food systems remains limited by technological constraints. High fiber content can negatively affect dough rheology, texture, and structural integrity, particularly at high inclusion levels [2,5]. It has been observed that at levels of 10% use of BSG in various products, the final structure increases, making it harder (more than 10%) and more cracked, and can interact with proteins, which reduces its extensibility (between 25 and 42%), gas retention, and water absorption capacity [2,3,4]. These challenges often result in reduced consumer acceptability and difficulties with large-scale processing. Therefore, the successful utilization of BSG requires an integrated approach that considers both formulation optimization and process feasibility to ensure product quality and scalability [6].

In addition to nutritional and sustainability drivers, market demand also supports the development of value-added snack products. The snack sector in Mexico represents a growing market, with increasing consumer interest in alternative products such as vegetable-based and functional snacks. Sweet potato-derived snacks, in particular, have gained attention due to their perceived health benefits and sensory appeal. This market context creates an opportunity to develop innovative snack formulations that integrate functional ingredients and agro-industrial by-products.

In this context, combining BSG with other ingredients that enhance processability and product stability is a viable strategy. Sweet potato is particularly relevant due to its content of carotenoids, antioxidants, and its functional role in improving matrix formation [7]. Likewise, cereal flours such as maize and wheat provide essential structural and technological properties that support mixing, shaping, and thermal processing operations. The integration of these ingredients enables the development of composite formulations that balance nutritional enhancement with process performance.

While significant research has focused on the formulation and characterization of functional snacks incorporating alternative ingredients, limited attention has been given to their techno-economic feasibility at the production scale. Most existing studies focus on product development and nutritional evaluation, with less attention to the economic and operational challenges of scaling up these formulations. In particular, there is a lack of studies integrating experimentally validated formulations with process simulation to assess both technical performance and economic viability.

Techno-economic analysis (TEA), supported by process simulation tools, provides a valuable framework for evaluating the scalability of food production systems. By integrating mass and energy balances with economic indicators, TEA enables the identification of process bottlenecks, estimation of production costs, and assessment of investment feasibility. This approach is particularly relevant for novel food products that involve complex formulations and multiple processing stages [8,9,10]. TEA has also been applied to valorize food industry side streams, thereby reducing operating costs and improving overall process sustainability [11].

Therefore, the objective of this study was to evaluate the techno-economic feasibility of producing a functional baked snack formulated with sweet potato flour, brewer’s spent grain, and cereals. The analysis is based on an experimentally optimized formulation, which is translated into a process simulation model using SuperPro Designer. The study integrates process design, scale-up analysis, and economic evaluation to identify key factors affecting production performance and profitability. Additionally, this work aims to contribute to the development of sustainable snack production systems by demonstrating the potential of agro-industrial by-products within economically viable processing frameworks.

Importantly, this study addresses a critical gap in the literature by integrating experimentally validated food formulations with process simulation and techno-economic assessment. While previous studies have predominantly focused on either product development or large-scale processing systems, this work provides a unified framework that connects laboratory-scale data with industrial feasibility for functional snack production. This approach offers a novel perspective for evaluating the scalability of multi-component food formulations within realistic processing and economic constraints.

## 2. Materials and Methods

### 2.1. Description of the Process Design

A production capacity of 1000 metric tons per year was selected as the basis for process design and simulation. This scale was strategically defined to align with the current dynamics of the Mexican functional snack market, where the sweet potato product segment is projected to reach USD 3110 million in valuation by 2025 [12]. Considering a target consumer price of 25.64 USD/kg, the total annual demand for this category is estimated at 121,295 metric tons (MT). Therefore, the selected throughput of 1000 MT/year represents an industrial foothold of approximately 0.8% of the total national demand. This capacity was defined to represent a small-to-medium industrial scale, allowing the evaluation of process performance under realistic production conditions while maintaining manageable operational complexity. The selected scale enables assessment of equipment requirements, batch scheduling, and economic feasibility within a production framework aligned with emerging functional snack products.

This production capacity also facilitates the analysis of process bottlenecks and the implementation of scale-up strategies, such as equipment staggering and cycle-time optimization, without introducing excessive uncertainty associated with large-scale industrial projections. Therefore, the selected throughput provides a suitable balance between technical feasibility and economic evaluation for simulation-based analysis.

The process was modeled in SuperPro Designer (v. 13, Intelligence, Inc., Scotch Plains, NJ, USA) by translating experimentally validated laboratory conditions into industrial-scale unit operations. The modeling followed a rigorous four-stage methodology to ensure that the simulation is grounded on an experimentally validated proof of concept prior to scale-up: (i) Model calibration: establishing mass and energy balances based on experimental stoichiometry and laboratory yields, the simulation was initialized by locking software input parameters to experimentally derived batch yields and residence times to ensure thermodynamic consistency. (ii) Process mapping: linking empirical results such as temperature, weight loss, mixing ratios, and residence times, with specific software input parameters. This stage ensures that the recipe in SuperPro accurately mirrors the laboratory protocol. (iii) Consistency Verification: Comparing simulation outputs against laboratory data to verify the model’s accuracy. This step is critical as it forms the basis for scaling up to the desired throughput; as noted in recent techno-economic studies, establishing a verified batch size and cycle time at a smaller scale is a prerequisite for industrial projections. (iv) Industrial Scaling and Debottlenecking: Expanding the validated baseline to the target throughput (1000 MT/year) through equipment staggering and cycle time optimization. It is important to note that stage (iii) does not represent a full model validation, as both the experimental and simulated data originate from the same laboratory-scale framework. Instead, this step serves as a consistency check to ensure that the simulation accurately reproduces the experimentally observed process behavior. A complete validation of the model would require pilot-scale or industrial data, which is beyond the scope of this study and is recommended for future work.

**Figure 1:**
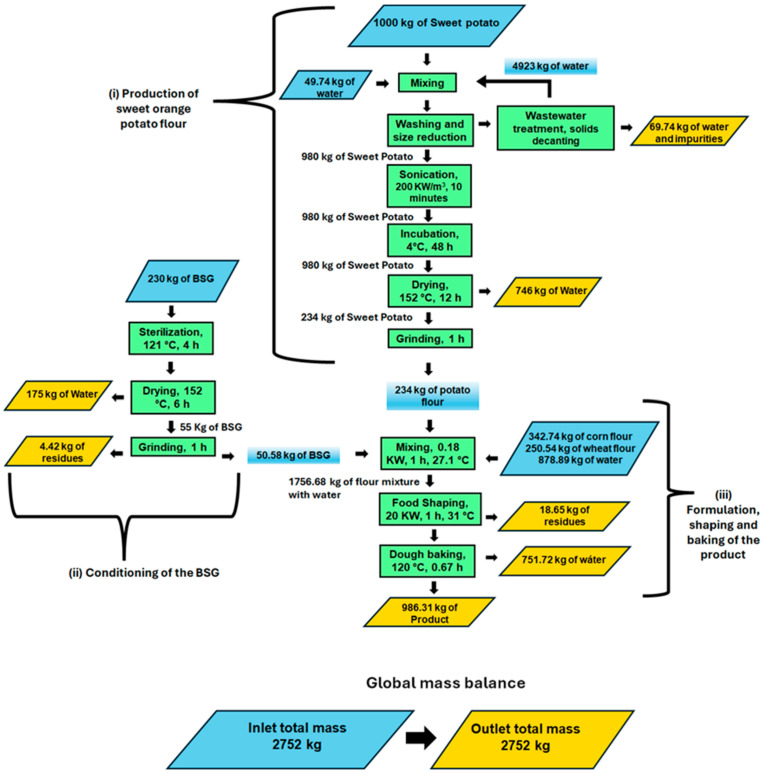
Mass balance of the entire process for producing sustainable snacks. The color coding is defined as follows: blue parallelograms represent process inputs (raw materials and water); yellow parallelograms represent process outputs (final product, residues, and water loss); green rectangles indicate unit operations; and gradient blue boxes represent internal process recirculations of intermediate materials.

To implement this methodology, a detailed Process Flow Diagram (PFD) was developed (Figure 1) that integrates the operational sequence and mass balances derived from the validated experimental conditions. The computational basis for the simulation was set at 1 MT of processed sweet potato per batch. The systematic transformation of these experimental conditions into software operations is further detailed in Table 1, which provides a point-by-point correspondence between the optimized laboratory parameters and their respective simulation inputs, with supporting references. Since SuperPro Designer lacks specific built-in models for complex food-shaping and industrial baking, these stages were modeled using Generic Boxes. These unit procedures were parameterized using validated technical specifications. The shaping equipment was simulated using technical details obtained online [13], including a capacity of 3000 pieces per hour, a power input of 20 kW, and a standard price of USD 22,000. The oven was simulated with a capacity of 200 kg/h, a power input of 4.8 kW, and a purchase price of USD 5000 [14].

**Figure 2:**
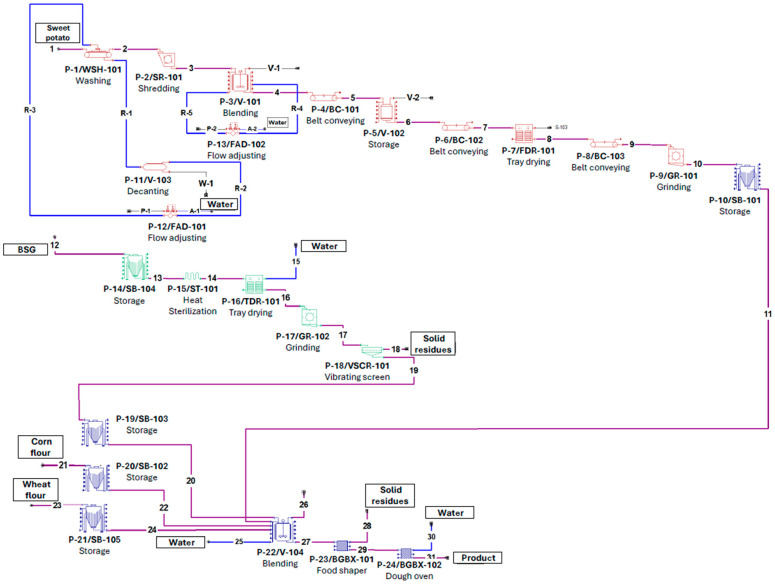
Flow diagram of the process done in SuperPro Designer V.13. Red icons represent the sweet potato flour production, green icons denote the BSG flour production, and blue icons signify the final product elaboration. The content and composition of each stream, represented by a number, can be further consulted in Table S8.

Once the process stages, unit procedures, mass balance, and input parameters are established, construction of the flow diagram in SuperPro Designer can begin. The resulting Process Flow Diagram (Figure 2) is divided into three functional stages: (i) production of orange sweet potato flour (red equipment), this stage includes six operations validated by previous experimental work [15]; (ii) conditioning of the BSG (green equipment) [16]. (iii) formulation, shaping, and baking of the product (blue equipment) [16]. In Stage 1 of wastewater treatment for sweet potato washing, sedimentation was employed to handle high volumes (up to 5 L/kg) and remove heavy loads of inert solids [17]. While simple settling achieves a ‘Satisfactory’ (40–60%) reduction in suspended solids, the process was enhanced with coagulation and flocculation to reach an ‘Excellent’ (>90%) efficiency rating [17]. Due to software licensing limitations that restricted the number of simulated units, the wastewater treatment stage was simplified to include only a sedimentation tank, allowing greater emphasis on other stages of the production process. However, to address the requirements for effective water reuse and compliance with food safety standards, the cost of purchasing unlisted equipment was included in the economic analysis to account for secondary treatments, such as UV disinfection. This additional treatment is critical for mitigating *Escherichia coli* contamination and ensuring the microbial safety of recycled water [18].

Also, in the sweet potato processing stage, raw material was first washed and subjected to ultrasound treatment to enhance mass transfer and improve functional properties. Following ultrasound treatment, the material was stored at 4 °C for 48 h. This operation was included based on previously validated experimental conditions, where this stage was required to stabilize the sweet potato matrix. The storage period promotes structural reorganization and moisture redistribution, which are critical for achieving consistent drying behavior and product quality. Although this step increases processing time, it was retained to preserve the experimentally validated process conditions and to evaluate its impact on production capacity during scale-up.

The BSG conditioning stage included drying, grinding, and sterilization operations. A sterilization step was incorporated due to the high initial moisture content and nutrient availability of BSG, which makes it particularly susceptible to microbial growth and contamination. In contrast, cereal flours used in the formulation were assumed to be commercially processed and microbiologically stable and therefore did not require additional sterilization within the modeled process.

The final stage consisted of ingredient blending, shaping, and baking. Due to software limitations in representing specific food-processing equipment, shaping and baking operations were modeled using generic unit procedures parameterized with industrial specifications sourced from commercial equipment.

### 2.2. Economic Assessment of the Designed Process

SuperPro Designer includes a preloaded template for performing an economic assessment of a given process flow diagram. The details of this set are provided elsewhere [19]; however, a brief description is as follows: the economic assessment assumes a 15-year project duration, with a 30-month construction period, a 4-month startup phase, and a 10-year depreciation period. The percentage of annual gross profits allocated to income taxes was fixed at 40%. Other costs were 13.8 USD/m^3^ for water, 0.1 USD/kWh for power, 4.83 USD/h for labor, 0.5 USD/MT for chilled water, 0.1 USD/MT for cooling water, 25 USD/MWh for natural gas, and 32 USD/MT for steam [20]. Due to software-specific equipment capacity limits, secondary downstream operations, such as packaging lines, were not explicitly modeled. However, their economic impact was included in the total annual operating costs under ‘Consumables.’ Additionally, marketing and advertising expenses were modeled as 5% of the total annual revenue, based on industry guidelines [21].

The cost of equipment, known as Purchase Cost (PC), was determined using a combination of internal software data and the factor-estimation methodology detailed in Table 2. The software estimates PC values using integrated cost functions that automatically adjust based on the year of analysis and the required capacity (e.g., tank volume or drying area) calculated in the mass balance for a given throughput.

The economic analysis conducted using SuperPro Designer was structured as a hierarchical classification of costs, as detailed in Table 2, enabling a transition from the acquisition of individual assets to the determination of the Total Capital Investment (TCI) and annual operating costs (AOC). This methodology has been reported elsewhere [22]. Firstly, the Fixed Capital Investment (FCI) is categorized into: (i) Direct Costs, which include equipment purchase costs (PC) and physical installation expenses such as piping, instrumentation, buildings, etc; (ii) Indirect Costs, which encompass engineering and construction; and (iii) Other Costs, allocated for contractors and contingencies. Based on this framework, the TCI was calculated by integrating Working Capital (WC)—estimated to cover 30 days of operation—and Startup Costs (SC), ensuring a comprehensive financial perspective of the project. Finally, the model defines Annual Operating Costs (AOC) as the sum of facility-dependent costs (maintenance and depreciation), consumables (raw materials and utilities), and labor. This factor-based estimation methodology ensures that both the initial investment required and recurring expenses are systematically and transparently accounted for.

### 2.3. Profitability Assessment of the Designed Process

The profitability of the project was evaluated using several economic indicators, as defined in the following equations [19].

(1) Gross profit=Revenues−Annual operating costs

(2) Net profit=Gross profit−Taxes+Depreciation

(3) $$Return\;of\;investment\;(ROI,\;\%)=\frac{Net\;Profit}{Total\;investment}$$

(4) $$Payback\;time\;(years)=\frac{Total\;investmant}{Net\;profit}$$

(5) $$NPV=\sum_{k=1}^{N}\frac{{NCF}_{k}}{{\left(1+i\right)}^{k}}$$

where *i* is the interest rate, *NCF_k_* is the net cash flow in year *k*, and *N* is the project lifetime (in years). The Net Present Value (*NPV*) is an indicator that enables comparisons among investment projects.

### 2.4. Sensitivity Analysis of the Process Design

The baseline scenario described in the flow process diagram (Figure 2) was subjected to a sensitivity analysis through the sensitivity coefficient (Equation (6)) to assess its economic and profitability impact [23].

(6) $$Sensitivity\;coefficient=\frac{∆Q_{i}}{∆P_{i}}$$

where ∆*P_i_* is the variation of the parameter analyzed, and ∆*Q_i_* represents the resulting variation in economic parameters (*NPV*, Internal rate of return (*IRR*), Return on Investment (*ROI*), Gross margin, and Payback time). The parameters analyzed using sensitivity coefficients include the final selling price, the snack’s production volume, and the costs of raw materials.

## 3. Results and Discussion

### 3.1. Techno-Economic and Sustainability Assessment

The base scenario constructed in SuperPro Designer was validated by ensuring it accurately replicated experimental data at critical process points, establishing a robust Proof of Concept before scale-up. This validation included (i) weight loss due to water evaporation during the drying stages of both sweet potato and Brewer’s spent grain (BSG), (ii) mass reduction during the baking of the final product, and (iii) the optimal mixing ratios of ingredients identified in the laboratory phase (see Figure 1). To ensure thermodynamic and operational consistency, a comparison between experimental yields and simulation outputs was performed, revealing deviations of less than 1% across all critical parameters. This agreement confirms that the simulation reliably reproduces the experimentally validated process behavior, supporting its use for subsequent scale-up analysis.

For this study, an annual operating time of 330 days was considered. The base simulation is characterized by a batch cycle time of 73.43 h and a specific output of 986.31 kg of final product per batch. However, it is possible to establish a minimum cycle time (*MCT*) of 50.32 h per batch by overlapping the start of a new batch with the time the previous batch is still in progress. This *MCT* is calculated based on the unit procedure with the longest duration (the process bottleneck), namely, storage at 4 °C for 48 h (P-3/V-101). Taking this minimum batch cycle time into account, a total of 156 batches per year can be produced. With this *MCT* value, the annual production rate in the base simulation is estimated at 154.32 MT/year. Since this baseline capacity is significantly lower than the strategic production target of 1000 MT/year, a systematic scale-up was performed through operational improvement rather than arbitrary equipment expansion. Process throughput was enhanced by addressing the primary bottleneck identified in the simulation: the refrigerated storage stage (P-3/V-102), which requires a 48 h residence time at 4 °C. To mitigate this constraint without increasing equipment scale, an equipment-staggering strategy was implemented in the SuperPro Designer interface. This mode allows multiple units to be deployed in parallel, enabling new batches to start before the preceding ones have cleared the bottleneck. Consequently, the plant’s batch frequency and total annual production increase by minimizing equipment underutilization and reducing the overall *MCT*.

**Figure 3:**
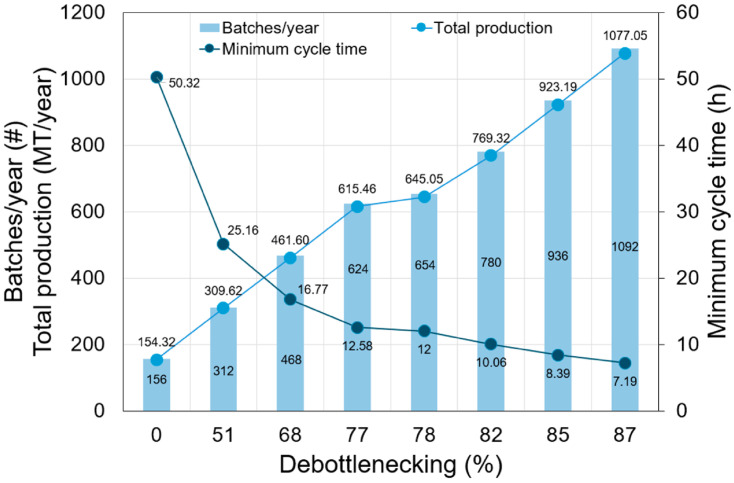
Effect of the process debottlenecking level on total production and batch configuration. # indicates number.

The efficiency of this debottlenecking strategy was quantified using the level of debottlenecking, as defined in Equation (7):

(7) $$Debottlenecking\;level\;(\%)=\frac{{MCT}_{i}-{MCT}_{f}}{{MCT}_{i}-{MCT}_{target}}\times 100$$

where *MCT_i_* and *MCT_f_* represent the initial and final minimum cycle times of the process, respectively. A theoretical target cycle time (*MCT_target_*) of 1 h was established. This value represents the vessel’s irreducible processing time, accounting for essential non-processing operations such as cleaning-in-place (*CIP*) and equipment charging. Conducting a debottlenecking analysis is critical for identifying and resolving constraints that limit production throughput and economic viability in complex batch processes [24]. The relationship between debottlenecking levels and annual production capacity is illustrated in Figure 3. The baseline scenario (0% debottlenecking) was initially limited by the refrigerated storage capacity (P-3/V-102). By implementing a staggering strategy with two parallel units, the debottlenecking level increased to 51%. This capacity expansion plateaued at four parallel units (77% debottlenecking), after which a bottleneck shift occurred: the drying stage (P-7/FDR-101) emerged as the new rate-limiting operation. To further improve throughput, a combined staggering strategy was deployed, utilizing seven storage units in parallel with two drying units. This strategy reduced *MCT* to 7.19 h, achieving an 87% debottlenecking level. Consequently, the facility reached a duty of 1092 batches per year, yielding a total annual production of 1077 MT. This configuration effectively synchronizes the capacities of the two most restrictive operations, successfully meeting the strategic production target while increasing overall line efficiency. This procedure of identifying “economic hotspots”, such as equipment capacity limits or lengthy cycle times, allows for increasing the frequency of batches and hence the annual production rate. So far, this strategy has been reported as a practical approach to increase plant productivity without expanding equipment size [25], which is particularly advantageous when local supplier limitations constrain equipment dimensions. In the context of the circular economy and the food industry, debottlenecking facilitates the integration of waste valorization strategies by resolving technical barriers that arise when scaling from lab to industrial levels, thereby minimizing resource use while maintaining high process efficiency [26]. Ultimately, removing these bottlenecks is essential for achieving a robust design that balances profitability with environmental sustainability [27].

Table 3 details the capital expenditure required for the production line, along with its specific characteristics, totaling a significant investment in both raw material processing and product finalization. The total capital investment for the main equipment is distributed across three functional stages, totaling USD 24,617,897. Stage 1, dedicated to Orange sweet potato flour production, represents the highest investment at USD 13,843,236, driven largely by the specialized storage needs of the V-102 receiver tanks (USD 497,000) and the FDR-101 tray dryers (USD 364,000). Stage 2, focused on BSG conditioning through sterilization and drying, requires an investment of USD 4,862,868, with the ST-101 heat sterilizer (USD 166,000) being the most expensive unit in this phase. Finally, Stage 3 involves the formulation, shaping, and baking of the product, with a total cost of USD 5,911,793, where the BGBX-101 shaping machine (USD 274,000) and the flour storage bins (SB-102 and SB-105) are the primary cost components. Regarding wastewater treatment, the sedimentation unit (V-103) was simulated for total solids removal near 99%, while water loss was fixed at 1% per batch. The total investment cost for wastewater treatment is up to USD 538,886. This investment reduces water consumption from 5506 m^3^/year (without reuse) to approximately 60 m^3^/year (with reuse). Although the operational cost of the WWT system (98,148 USD/year) is higher than the cost of sourcing untreated freshwater (76,000 USD/year), this investment is technically and environmentally justified. Consequently, higher expenditure represents a transition toward a ‘circular water economy,’ mitigating the environmental impact of high-load effluents and ensuring process resilience against increasing water scarcity and stricter environmental regulations [17,18].

Flour is a crucial ingredient in the food industry; its commercialization requires several metric tons annually to be economically viable. For example, Olivera-Montenegro et al. (2022) [28] reported a higher-capacity model for defatted quinoa flour of up to 269,998 MT/year, while [8] used SuperPro Designer for VitAto sweet potato flour with a capacity of 32,000 MT/year. The biorefinery concept supports these scales, integrating flour production with other value-added streams. Solarte-Toro et al. (2025) [29] described a 1200 MT/year plant for Sacha Inchi, while [30] evaluated plantain flour and bioethanol production, improving process performance and nutritional content with a production scale of 1113.11 MT/day of corn for ethanol. In the present study, the designed process maintains a production rate of 1077 MT/year. While this capacity is at the lower range of reports found in the literature, it is strategically sized to capture at least 0.8% of the specific market for sweet potato-based snacks. This scale is consistent with techno-economic studies that focus on regional valorization and high-value niche products rather than on bulk commodity processing.

The total capital investment is predominantly driven by direct costs associated with plant construction, which account for approximately 50% of the total across all debottlenecking levels. This is followed by indirect costs (around 30%) and other costs, including the contractor’s fee and contingency, which account for approximately 20%. As expected, increasing annual production results in higher investment costs (up to 38%) and operating costs (up to 52%). However, there is also a significant decrease in unit production costs (up to 238%) (Table 4). Within the scenario with the maximum annual production reached at 85% of debottlenecking, the investment cost distribution per stage of the process is 56.23% to produce orange sweet potato flour (stage 1), 19.75% for the conditioning of the BSG (stage 2), and 24.01% for formulation, shaping, and baking of the product (stage 3).

**Figure 4:**
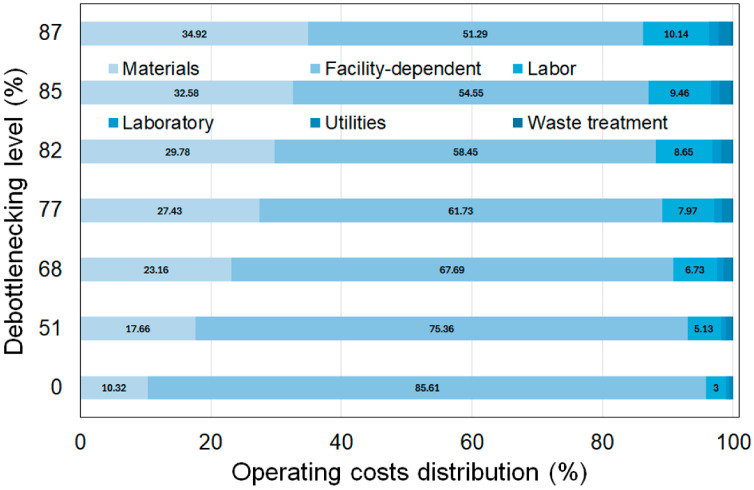
Distribution of the operating costs at the different configurations of the process.

The distribution of operating costs indicates that facility-dependent costs are the most significant (See Figure 4). These facility-dependent costs include equipment depreciation, maintenance, and insurance, among others. As the number of bottleneck equipment units increases, the percentage of facility-dependent costs decreases (up to 51.29%), while other costs, such as material (up to 34.92%) and labor (up to 10.14%), become more significant. Significantly, the expansion also affects utility costs (energy and water), which are categorized under operating expenses. The annual utility expenditure increased from USD 0.02218 million at the initial scale to USD 0.15374 million in the final scenario of 1077.21 MT produced annually. A detailed analysis of the distribution of the total operating cost for each stage of the scenario with a debottlenecking level of 87% (8.494 million USD/year) shows 66.51% to produce orange sweet potato flour (stage 1), 11.6% for the conditioning of the BSG (stage 2), and 21.89% for formulation, shaping, and baking of the product (stage 3). The utilities costs in this process encompass electricity, steam, cooling water, chilled water, freon, and natural gas. The detailed cost breakdown by utility type and stage is presented in Tables S1–S7. The total annual utility expenditure is estimated at 153,902 USD/year, with 80.85% allocated to orange sweet potato flour production (stage 1), 5.54% to BSG conditioning (stage 2), and 13.61% for formulation, shaping, and baking of the final product (stage 3). The annual utility cost is predominantly driven by energy expenditure, which totals 152,835.35 USD/year (Table 5). This cost is primarily distributed among the requirements for electricity, steam, and natural gas. As highlighted, Stage 1 is identified as the most energy-intensive phase of the process, with electricity costs amounting to 93,261.84 USD/year, representing most of the total electricity expenditure of 96,797.28 USD/year (Table 5). Within Stage 1, the ultrasound treatment and sweet potato drying are the most energy-demanding unit operations, collectively accounting for 67.77% of the total electricity cost. In industrial food processing, ultrasound-assisted treatments are known for their high power requirements, as they require energy to induce acoustic cavitation in dense vegetable slurries. According to industrial reports and literature on emerging technologies, although sonication significantly improves product quality, it remains an energy-intensive operation [31]. This explains the disproportionate impact of Stage 1 on the overall utility balance, a common trade-off when implementing high-intensity physical treatments to enhance the functional properties of root-based flours. To improve the economic profile of this stage, strategies such as heat integration for the drying units and optimized ultrasound pulsing should be considered. The dominance of Stage 1 in the utility profile is consistent with other root-based flour production models. For instance, in the production of VitAto (a Malaysian orange sweet potato variety), similar energy bottlenecks are observed during the initial processing stages required to convert raw tubers into stable flour [8].

**Figure 5:**
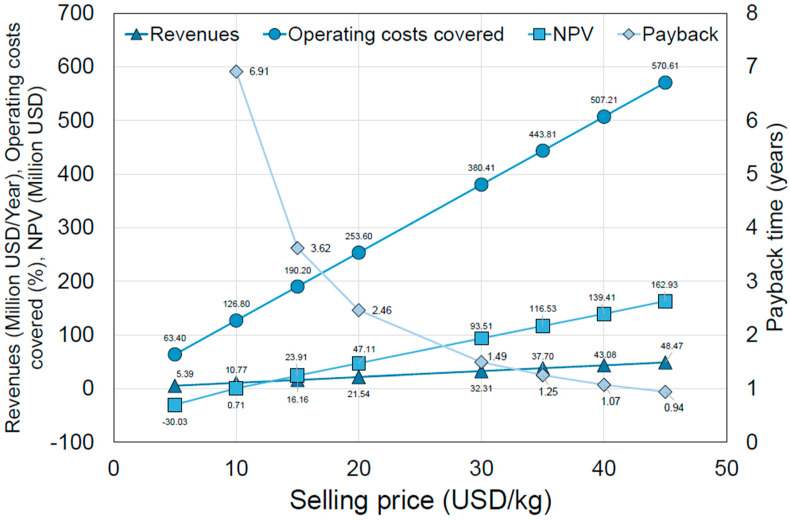
Impact of the selling price on several economic indicators.

**Figure 6:**
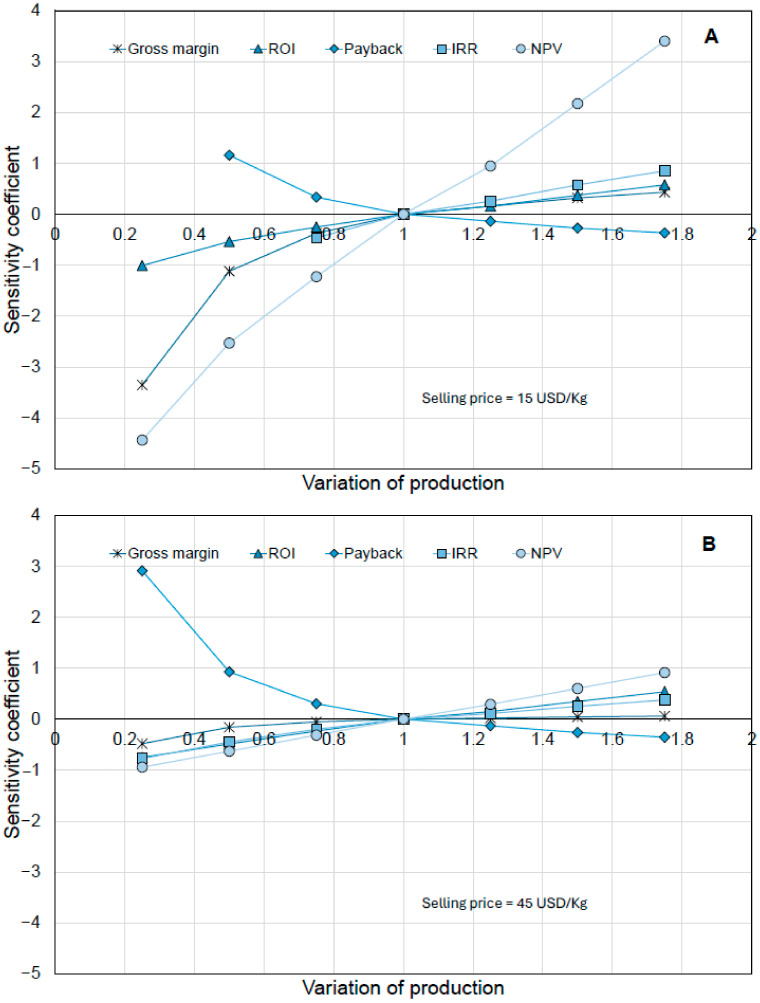
Sensitivity analysis for the selling price at different values of production. (**A**) Sensitivity coefficient for production variation at a selling price of 15 USD/kg (**B**) Sensitivity coefficient for production variation at a selling price of 45 USD/kg.

The scenario with the highest production, 1077 MT/year, was used to analyze the selling price and determine the profitability of the designed process (see Figure 5). For this analysis, a range of 5–45 USD/kg of product was considered, and its impact on several economic and financial indicators, including revenues, *NPV*, and payback time, was examined. The selected price range was initially used to explore process sensitivity across a wide range of economic scenarios. However, based on current market data for comparable snack products, a more realistic commercial range lies between approximately 8–25 USD/kg, depending on product positioning (e.g., conventional versus functional or premium snacks). Within this context, the proposed selling price of 15 USD/kg falls within the expected range for functional snack products and represents a balanced scenario between profitability and market competitiveness. For instance, Boravelli et al. (2026) [32] reported a minimum selling price (*MSP*) of 43.49 USD/kg for purified red beet colorants, whereas Rodríguez-Martínez et al. (2026) [33] identified an *MSP* of 3.77 USD/kg for succinic acid from potato peels. Our break-even price of 9.845 USD/kg falls between these benchmarks, reflecting the intermediate complexity of a multi-component functional snack. Higher price values (e.g, 45 USD/kg) are therefore considered as theoretical upper bounds used for sensitivity analysis rather than realistic market targets. Additionally, the percentage of operating costs covered by the revenues was calculated. At the lowest selling price, the *NPV* is negative; the payback period cannot be calculated, and operating costs are covered by revenues of only 63.4%, indicating a non-profitable scenario. As the selling price rises above 10 USD/kg, economic indicators become more optimistic. Break-even analysis was conducted to determine the point at which the *NPV* equals zero. This analysis identified a minimum selling price of 9.845 USD/kg for the Snack. This price includes the total capital investment and financing costs over the project’s lifetime, whereas unit operating costs cover only ongoing manufacturing expenses. The calculated break-even price serves as an essential benchmark for evaluating market sensitivity and investment risk [34]. At the proposed price of 15 USD/kg, the project yields a payback period of less than 5 years and a positive *NPV*, which is a standard requirement for industrial feasibility in the food sector [35]. It is important to note that *NPV* should be considered, as it determines the process’s profitability; it should be positive, although there is limited information on the optimal magnitude. At first glance, the *NPV* should at least match the investment value, which, in this scenario, is USD 28 million, corresponding to a selling price of 15–20 USD/kg. A selling price of 45 USD/kg yields the highest *NPV* of USD 162.93 million, which could be attractive to investors; however, this price may price the project out of the market if it is too high relative to competitors. Furthermore, the sensitivity analysis (Figure 6) reveals that the *NPV* is highly susceptible to production fluctuations. Variations in production may arise not only from process configurations but also from productivity declines resulting from equipment failures, defective batches, or unmet sales targets. At 15 USD/kg, a 25% decrease in production results in a 122% reduction in *NPV*, highlighting a ‘fragility’ observed in other biorefinery models. Pereira et al. (2025) [36] similarly noted that profitability in bioactive compound extraction is highly dependent on selling price and productivity, with payback times only becoming favorable in optimized large-scale scenarios.

In contrast, when the selling price is increased to 45 USD/kg, a 25% decrease in production leads to a 31.25% reduction in *NPV*. According to Figure 6A,B, the sensitivity ranking of the parameters, in order of importance, is as follows: *NPV* > Gross Margin > *ROI* > *IRR* > Payback Time. It is important to note that fluctuations in production can also be interpreted as changes in sales, which may disrupt the project’s financial planning. The sensitivity ranking observed (*NPV* > Gross Margin > *ROI* > *IRR* > Payback Time) underscores that production volume and market price are the most critical variables for the project’s financial stability. This prioritization is consistent with previous findings where feedstock costs and production capacity were identified as the primary drivers of manufacturing costs in potato-based biorefineries and betanin production [32,33].

**Figure 7:**
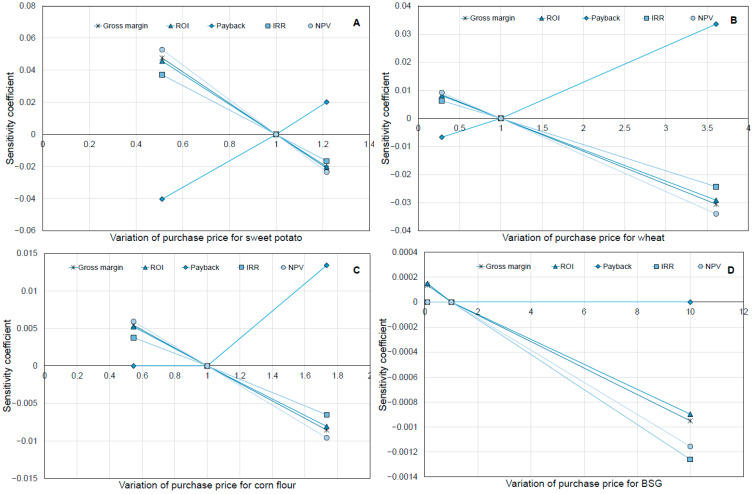
Sensitivity analysis for the effect of the selling price of the raw materials on the economic indicators. (**A**) Sensitivity coefficient for the purchase cost of sweet potato. (**B**) Sensitivity coefficient for the purchase cost of wheat. (**C**) Sensitivity coefficient for the purchase cost of corn flour. (**D**) Sensitivity coefficient for the purchase cost of BSG.

The sensitivity analysis was extended to account for variations in the raw materials used in the process. It is important to mention that, for the scenario with maximum production of snacks, which yields 1077 MT/year of snacks, 251 MT/year of BSG, 374.27 MT/year of corn flour, 273.59 MT/year of wheat flour, and 1070 MT/year of sweet potatoes are required. During the sensitivity analysis, a variation in the purchase price from 1.11 to 2.64 USD/kg was considered for sweet potato [37,38], a variation from 0.41 to 1.3 USD/kg for corn flour [39,40], and from 0.29 to 3.68 USD/kg for wheat flour [41,42], taking into account actual international market prices for these raw materials. Figure 7A shows that the purchase price of sweet potatoes exhibits the highest sensitivity coefficients across the economic and financial indicators. This result is expected, as sweet potatoes account for the largest share of raw material inputs and are the most expensive among the manufacturing process inputs. Although each raw material was evaluated over a different price range, the key observation is that the magnitudes of the sensitivity coefficients remain considerably lower than those shown in Figure 7, particularly for corn and whey flour (Figure 7B,C). Estimating the purchase price of BSG was particularly challenging because it depended on brewery disposal practices and transportation costs. Therefore, a wide variation, from 1 USD/MT to 100 USD/MT, was applied to assess its impact on the sensitivity coefficient. As shown in Figure 7D, even this broad price range had only a minimal effect on the sensitivity coefficient compared with the other raw materials, which may be advantageous when negotiating the acquisition of brewing industry residues.

The ranking of the raw materials, in order of importance affecting the financial parameters, is as follows: Sweet potato flour > wheat flour > corn flour > BSG.

Beyond techno-economic performance, implementing the proposed process requires consideration of regulatory, safety, and process-related constraints.

### 3.2. Regulatory and Process Safety Considerations

While the techno-economic analysis demonstrates the feasibility of the proposed process, its industrial implementation requires compliance with applicable regulatory and safety standards. In the context of the formulated snack, particular attention must be given to the use of BSG, which in some jurisdictions may be classified as a novel or non-traditional food ingredient. Consequently, pre-market authorization and compliance with food safety regulations are required, including proper allergen labeling due to its barley origin [43].

From a process perspective, safety considerations are closely linked to raw material quality and processing conditions. The drying and storage stages identified as critical operations in the simulation may also act as points where contaminants could concentrate or microbial growth could occur if not properly controlled. Therefore, implementing quality assurance strategies such as Hazard Analysis and Critical Control Points (*HACCP*), control of water activity, and strict temperature management is essential to ensure product safety and stability. Additionally, the presence of potential contaminants such as mycotoxins, pesticide residues, and heavy metals in raw materials must be monitored, particularly considering the concentration effects associated with dehydration processes. The factors should be incorporated into quality control protocols on an industrial scale.

Overall, integrating regulatory compliance and process safety considerations into the design and operation of the production system is essential to ensure successful commercialization and consumer safety. Additional details on regulatory aspects and safety considerations are provided in the Supplementary Materials.

### 3.3. Process Limitations, Product Constraints, and Future Work

The formulation used in this study reflects a balance between ingredient functionality and process feasibility. While higher levels of BSG could enhance the functional profile of the product, they may also introduce processing challenges that affect large-scale production.

The limitations discussed in this section refer to the overall formulation and process, rather than to a single ingredient. Despite the favorable techno-economic performance observed in this study, several process and product-related limitations must be considered for successful industrial implementation. From a process engineering perspective, the simulation identified storage and drying operations as critical bottlenecks that strongly influence production capacity and energy consumption. In particular, the 48 h refrigerated storage stage and the energy-intensive drying steps pose operational constraints that may affect scalability, process efficiency, and the overall cost structure. Future work should explore alternative processing strategies, such as reducing residence times, implementing continuous or semi-continuous configurations, and improving energy integration to enhance process efficiency.

From a product perspective, incorporating fiber-rich ingredients introduces formulation changes affecting texture, color, and structural properties. High fiber content can increase product hardness and affect matrix formation, while thermal processing may intensify browning reactions, influencing consumer perception. While these changes influence consumer perception, the process model developed here is strictly based on previously validated experimental data, ensuring that mass and energy balances reflect the physical reality of the formulation. The existing laboratory-scale parameters provide a sufficient and reliable baseline for establishing economic viability and operational constraints; therefore, additional experimental testing was considered outside the scope of this work, which focuses on the computational translation of laboratory innovation into a scalable framework. While future pilot-scale implementation could allow for fine-tuning sensory attributes or specific equipment configurations, the current simulation provides a robust assessment of the process’s industrial feasibility.

Future research should also focus on evaluating product stability, shelf life, and functional properties, as well as assessing regulatory compliance and market acceptance under real commercialization scenarios. Addressing these aspects will be critical for translating the proposed process from simulation to full-scale industrial application.

Beyond economic feasibility, this study presents a significant innovation for the functional food sector by developing a nutrient-dense snack that addresses current societal demands for health and sustainability. The novelty lies in the synergistic formulation of sweet potato and upcycled brewer’s spent grain, which not only enhances the fiber and antioxidant profile of the product but also offers a scalable solution for industrial by-product valorization.

Our main results demonstrate that the process is highly resilient, achieving a Net Present Value (*NPV*) of USD 23.908 million even when accounting for environmental management costs. Comparative analysis against current market standards shows that this formulation offers a competitive advantage in the production cost-to-nutrient ratio. These findings contribute to society by offering a viable model for small- to medium-sized enterprises to produce affordable, high-value functional foods, thereby supporting regional food systems and reducing industrial waste. To ensure full transparency and reproducibility of these findings, a comprehensive mass balance for every process stream and the detailed itemization of utility costs (including electricity, steam, and water consumption for each unit operation) are provided in the Supplementary Materials. These datasets support the summary figures presented in the main text and offer a rigorous foundation for the calculated operating expenses and overall process efficiency.

## 4. Conclusions

The techno-economic feasibility of producing a functional baked snack was demonstrated through an experimentally grounded process simulation approach. By integrating laboratory-scale validated parameters into SuperPro Designer, a consistent framework was established to bridge the gap between proof of concept and industrial-scale production. The model accurately reproduced key process variables and identified critical bottlenecks, particularly in storage and drying operations, which strongly influence production capacity and energy demand.

Through the implementation of an equipment-staggering strategy and cycle-time optimization, production capacity was successfully increased to 1077 MT/year without relying on arbitrary equipment scaling, improving operational efficiency and reducing unit production costs. Economic evaluation identified a break-even price of 9.85 USD/kg and a competitive selling price of 15 USD/kg, demonstrating the viability of the proposed process under realistic market conditions.

Beyond techno-economic performance, the results highlight the importance of considering formulation design, process constraints, and regulatory requirements when scaling up food production systems. In particular, the selected multi-component formulation reflects a balance between ingredient functionality and process feasibility, enabling stable operation while maintaining product performance.

Overall, this work provides a robust and transferable framework for evaluating the industrial feasibility of snack production systems, grounded in experimentally validated data. This approach advances the integration of formulation design and process simulation in the development of scalable food products, supporting the transition from laboratory innovation to industrial implementation. Future work should focus on pilot-scale validation, process optimization, and the assessment of product stability and regulatory compliance under real manufacturing conditions to support successful commercialization.

## Figures and Tables

**Table 1:** Technical correspondence table between experimental data and model input.

Process Stage	Unit Procedure in SuperPro	Operational Parameter (Validated Value)	Reference
Pre-treatment of sweet potato	Washing	5 L of water with 0.001% of sodium hypochlorite per kg of sweet potato T = 25 °C t = 2 h	[15]
Sonication of sweet potato	Blending tank	T = 25 °C t = 0.167 h Power input = 200 kW/m^3^	[15]
Incubation of sweet potato	Storage tank	T = 4 °C t = 48 h	[15]
Drying of sweet potato	Tray drying	Weight loss = 76.13% Steam inlet temperature 152 °C t = 12 h	[15]
Sterilization of Brewer’s spent grain (BSG)	Heat sterilization	T = 121 °C t = 4 h	[16]
Drying of BSG	Tray drying	Steam inlet temperature = 152 °C t = 6 h Final moisture content = 6.2%	[16]
Ingredients blending	Blending tank	t = 1 h Specific power input = 0.1 kW/m^3^ Recipe (% *w*/*w*): water (50), sweet potato flour (13.31), BSG (2.94), corn flour (19.50), wheat flour (14.25).	[16]
Food shaping	Generic box	Power input = 20 kW 3000 pieces/h 1% of losses	This work
Dough baking	Generic Box	T = 120 °C t = 40 min Natural gas Heating rate 1 °C/min Weight loss = 85.5%	This work

**Table 2:** Steps followed for economic analysis in SuperPro Designer.

Parameter	Value/Calculation
Purchase Cost (PC)	Listed Equipment Cost * + Unlisted Equipment Purchase Cost **
Unlisted Equipment Purchase Cost	0.2 * Purchase Cost
Direct Cost (DC) [21]	PC + Installation + PC * (A + B + C + D + E + F + G) Installation = 50% of PC A = Piping = 0.35 B = Instrumentation = 0.4 C = Insulation = 0.03 D = Electrical facilities = 0.1 E = Buildings = 0.45 F = Yard improvements = 0.15 G = Auxiliary facilities = 0.4
Indirect Costs (IC) [21]	Engineering (25% of DC) + Construction (35% of DC)
Other Costs (OC) [21]	Contractor (5% of DC + IC) + Contingency (10% of DC + IC)
Fixed Capital Investment (FCI)	DC + IC + OC
Working Capital (WC)	30 days of labor, raw material, utilities, and waste treatment
Startup Cost (SC)	5% of DFC
Total Capital Investment (TCI)	FCI + WC + SC
Annual Operating Costs (AOC)	Facility-dependent costs + Labor costs + Utility costs + Miscellaneous expenses + Lab/QC/QA costs
Facility-dependent Costs	Maintenance (6% of FCI) + Equipment Depreciation + Insurance (1% of FCI) + Local Taxes (2% of FCI) + Factory Expenditures (5% of FCI)
Total Labor Cost (TLC)	Labor Demand per Rate * Labor Rate per Type
Labor Rate per Type	Basic Labor Rate + Benefits (40%) + Supervision (20%) + Administration (60%) + Operating Supplies (10%)
Lab, QC, and QA Costs	15% of Total Labor Cost (TLC)

* Listed equipment includes operation units shown in the flow diagram. ** Unlisted equipment includes smaller units not shown but accounted for as a percentage of total equipment cost.

**Table 3:** List of main equipment and their characteristics determined for the production of the snack.

Name	Stage of Process	Type	Purpose	Ocuppancy Time (% of Recipe Cycle Time)	Size (Capacity)	Units	Unit Price (USD/Unit)	Total Price (USD)
WSH-101	1 (Orange Sweet potato flour)	Washer	Washing of the raw sweet potato with 5 L of water per kg	2 h (27.82%)	500	1	6500	6500
SR-101	1 (Orange Sweet potato flour)	Shredder	Machine for slicing potatoes	1 h (13.91%)	980.00 kg/h	1	108,000	108,000
V-101	1 (Orange Sweet potato flour)	Blending Tank	Tank for ultrasound treatment of potatoes	4.42 h (61.44%)	6090.06 L	2	123,000	246,000
BC-101, 102 and 103	1 (Orange Sweet potato flour)	Belt conveyor	Transport of solids	1.25 h (17.39%)	1200 kg/h	3	75,000	225,000
V-102	1 (Orange Sweet potato flour)	Receiver Tank	Tank for storage at 4 °C of potatoes for 48 h	7.19 h (100%) *	1087.12 L	7	71,000	497,000
FDR-101	1 (Orange Sweet potato flour)	Tray Dryer	Drying of potatoes	6 h (83.47%) *	65.37 m^2^	2	182,000	364,000
GR-101	1 (Orange Sweet potato flour)	Grinder	Grinding of potatoes	1 h (13.91%)	233.93 kg/h	1	104,000	104,000
SB-101	1 (Orange Sweet potato flour)	Solids Bin	Storage of the sweet potato flour	1.25 h (17.39%)	344.01 L	1	158,000	158,000
V-103	1 (Orange Sweet potato flour)	Decanter Tank	Decanting tank for impurity removal	1 h (13.91%)	674.92 L	1	66,000	66,000
SB-104	2 (BSG conditioning)	Solids Bin	Tank for reception and storage of fresh BSG	1.38 h (19.24%)	338.24 L	1	156,000	156,000
ST-101	2 (BSG conditioning)	Heat Sterilizer	Sterilization of BSG	4 h (55.65%)	57.12 L/h	1	166,000	166,000
TDR-101	2 (BSG conditioning)	Tray Dryer	Drying of BSG	6 h (83.47%)	11.58 m^2^	1	113,000	113,000
GR-102	2 (BSG conditioning)	Grinder	Grinding of dried BSG	1 h (13.91%)	55.00 kg/h	1	103,000	103,000
VSCR-101	2 (BSG conditioning)	Vibrating Screen	Screening of the BSG flour	1 h (13.91%)	55.00 kg/h	1	8000	8000
SB-103	2 (BSG conditioning)	Solids Bin	Tank for storage of finished BSG flour	1 h (13.91%)	74.38 L	1	75,000	75,000
SB-102	3 (Formulation, shaping, and baking of product)	Solids Bin	Tank for storage of corn flour	1.25 h (17.39%)	504.03 L	1	199,000	199,000
SB-105	3 (Formulation, shaping, and baking of product)	Solids Bin	Tank for storage of wheat flour	1.25 h (17.39%)	368.44 L	1	165,000	165,000
V-104	3 (Formulation, shaping, and baking of product)	Blending Tank	Mixing tank for formulation snacks	2.5 h (34.78%)	1955.13 L	1	104,000	104,000
BGBX-101	3 (Formulation, shaping, and baking of product)	Batch Generic Box	Machine for Shaping Snacks	3.28 h (45.62%)	1956.55 L	1	274,000	274,000
BGBX-102	3 (Formulation, shaping, and baking of product)	Batch Generic Box	Oven for baking snacks	4.56 h (63.45%)	1957.31 L	1	13,000	13,000

* These values represent the effective equipment occupancy time divided by the total number of equipment sets in staggered mode.

**Table 4:** Detailed cost data for different levels of Debottlenecking.

Debottlenecking Level (%)	0	51	68	77	82	85	87
Total equipment cost (Million USD)	3.169	3.258	3.347	3.436	3.752	3.841	3.929
Total direct cost (Million USD)	10.134	10.419	10.705	10.991	11.982	12.268	12.553
Total indirect cost (Million USD)	6.080	6.252	6.423	6.595	7.189	7.361	7.532
Other cost (Million USD)	2.432	2.501	2.569	2.638	2.876	2.944	3.013
Working capital (Million USD)	0.052	0.104	0.156	0.208	0.261	0.313	0.365
Start-up cost (Million USD)	0.932	0.959	0.985	1.011	1.102	1.129	1.155
Total capital investment (Million USD)	19.63	20.235	20.839	21.443	23.41	24.014	24.618
Total operating cost (Million USD)	4.108	4.798	5.488	6.178	7.114	7.804	8.494
Unit production cost (USD/kg)	26.7	15.59	11.89	10.04	9.25	8.46	7.89

**Table 5:** Energy Consumption and Cost Distribution by Equipment and Source.

Electricity				
Unit Procedure	Unit Cost (USD/kWh)	Amount (kWh/year)	Cost (USD/year)	%
WSH-101, Washing of the raw sweet potato	0.10	10,920	1092.00	1.13
SR-101, Machine for slicing potatoes	0.10	53,508	5350.80	5.53
V-101, Tank for ultrasound treatment of potatoes	0.10	399,021	39,902.06	41.22
BC-101, Transport of solids	0.10	54	5.35	0.01
BC-102, Transport of solids	0.10	54	5.35	0.01
FDR-101, Drying of sweet potatoes	0.10	256,982	25,698.16	26.55
BC-103, Transport of solids	0.10	13	1.28	0.001
GR-101, Grinding of potatoes	0.10	25,545	2554.47	2.74
TDR-101, Drying of BSG	0.10	6006	600.61	0.62
GR-102, Grinding of dried BSG	0.10	53	5.34	0.01
V-104, Mixing tank for formulation snacks	0.10	192	19.21	0.02
BGBX-101, Machine for Shaping Snacks	0.10	21,936	2193.61	2.27
BGBX-101, Oven for baking snacks	0.10	96	9.57	0.01
Unlisted Equipment	0.10	48,399	4839.86	5.00
General load	0.10	145,196	14,519.60	15
**TOTAL**		**967,972**	**96,797.28**	**100.00**
**Steam**				
**Unit procedure**	**Unit cost (USD/MT)**	**Amount (MT/year)**	**Cost (USD/year)**	**%**
FDR-101, Drying of sweet potatoes	32.00	974	31,159.04	80.23
ST-101, Sterilization of BSG	32.00	13	409.84	1.06
TDR-101, Drying of BSG	32.00	227	7269.55	18.72
**TOTAL**		**1214**	**38,838.43**	**100**
**Natural gas**				
**Unit procedure**	**Unit cost (USD/MWh)**	**Amount (MWh/year)**	**Cost (USD/year)**	**%**
BGBX-101, Oven for baking snacks	25.00	688	17,199.64	100.00

## Data Availability

The original contributions presented in this study are included in the article/Supplementary Materials. Further inquiries can be directed to the corresponding author.

## Supplementary Materials

The following supporting information can be downloaded at https://www.mdpi.com/article/10.3390/foods15101654/s1, Table S1: Utilities cost, electricity; Table S2: Utilities cost, steam; Table S3: Utilities cost, cooling water; Table S4: Utilities cost, chilled water; Table S5: Utilities cost, freon; Table S6: Utilities cost, natural gas; Table S7: Detailed breakdown by utility type and unit procedure; Table S8: Mass balance of the Process flow diagram (Figure 3). Supplementary Material File S2: Extended Discussion on Regulatory Aspects and Product-Related Considerations. References [2,4,43,44,45,46,47,48,49,50,51,52,53,54,55,56,57,58,59,60] are cited in the Supplementary Materials.

## Abbreviations

The following abbreviations are used in this manuscript:

AOC	Annual Operating Costs
BSG	Brewer’s Spent Grain
CAPEX	Capital Expenditure
CIP	Cleaning-in-Place
DC	Direct Costs
FCI	Fixed Capital Investment
IRR	Internal Rate of Return
MCT	Minimum Cycle Time
NPV	Net Present Value
OPEX	Operating Expenditure
PFD	Process Flow Diagram
ROI	Return on Investment
SC	Startup Cost
TEA	Techno-Economic Analysis
TLC	Total Labor Cost
WC	Working Capital
WT	Water Treatment
QC	Quality Control
QA	Quality Assurance
