# Supplementary material for: Techno-Economic Feasibility of Functional Snacks from Brewer’s Spent Grain and Sweet Potato: A Simulation Study
Source: Foods. 2026 May 9;15(10):1654. doi: 10.3390/foods15101654 (PMC13205998; doi:10.3390/foods15101654)
Supplement: Supplementary file 1 [file foods-15-01654-s001.zip › Suplementary material 1-TEA.pdf]

Table S1. Utilities cost: electricity

Stage	Unit cost (USD/KW-h)	Amount (KW-h/year)	Cost (USD/year)	%
Stage 1: Orange Sweet Potato flour)	0.1	932,618	93,261.84	96.35
Stage 2: Conditioning of the BSG	0.1	7,574	757.44	0.78
Stage 3: formulation, shaping, and baking of the final product	0.1	27,780	2,778.00	2.87
TOTAL		967,973	96,797.28	100.00

Table S2. Utilities cost: steam

Stage	Unit cost (USD/MT)	Amount (MT/year)	Cost (USD/year)	%
Stage 1: Orange Sweet Potato flour)	32	974	31,169.29	54.58
Stage 2: Conditioning of the BSG	32	240	7,765.45	13.60
TOTAL		1,214	57,104.92	100.00

Table S3. Utilities cost: cooling water

Stage	Unit cost (USD/MT)	Amount (MT/year)	Cost (USD/year)	%
Stage 2: Conditioning of the BSG	0.1	861	86.06	100.00
TOTAL		861	86.06	100.00

Table S4. Utilities cost: chilled water

Stage	Unit cost (USD/MT)	Amount (MT/year)	Cost (USD/year)	%
Stage 3: formulation, shaping, and	0.5	1,941	970.54	100.00

baking of the final product				
TOTAL		1,941	970.54	100.00

Table S5. Utilities cost: freon

Stage	Unit cost (USD/MT)	Amount (MT/year)	Cost (USD/year)	%
Stage 1: Orange Sweet Potato flour)	0.15	68	10.26	100.00
TOTAL		68	10.26	100.00

Table S6. Utilities cost: natural gas

Stage	Unit cost (USD/ MW-h)	Amount (MW-h /year)	Cost (USD/year)	%
Stage 3: formulation, shaping, and baking of the final product	25	688	17,199.64	100.00
TOTAL		688	17,199.64	100.00

Table S7. Detailed breakdown by utility type and unit procedure.

ELECTRICITY				
**Unit procedure**	**Unit cost (USD/KW-h)**	**Amount (KW-h/year)**	**Cost (USD/year)**	**%**
WSH-101; Washing of the raw sweet potato	0.10	10,920	1,092.00	1.13
SR-101; Machine for slicing potatoes	0.10	53,508	5,350.80	5.53
V-101; Tank for ultrasound treatment of potatoes	0.10	399,021	39,902.06	41.22
BC-101, Transport of solids	0.10	54	5.35	0.01
BC-102, Transport of solids	0.10	54	5.35	0.01
FDR-101, Drying of sweet potatoes	0.10	256,982	25,698.16	26.55
BC-103, Transport of solids	0.10	13	1.28	0.001
GR-101, Grinding of potatoes	0.10	25,545	2,554.47	2.74
TDR-101, Drying of BSG	0.10	6,006	600.61	0.62
GR-102, Grinding of dried BSG	0.10	53	5.34	0.01
V-104, Mixing tank for formulation snacks	0.10	192	19.21	0.02
BGBX-101, Machine for Shaping Snacks	0.10	21,936	2,193.61	2.27
BGBX-101, Oven for the baking snacks	0.10	96	9.57	0.01

Unlisted Equipment	0.10	48,399	4,839.86	5.00
General load	0.10	145,196	14,519.60	15
**TOTAL**		**967,972**	**96,797.28**	**100.00**
**STEAM**				
**Unit procedure**	**Unit cost (USD/MT)**	**Amount (MT/year)**	**Cost (USD/year)**	**%**
FDR-101, Drying of sweet potatos	32.00	974	31,159.04	80.23
ST-101, Sterilization of BSG	32.00	13	409.84	1.06
TDR-101, Drying of BSG	32.00	227	7,269.55	18.72
**TOTAL**		**1,214**	**38,838.43**	**100**
**COOLING WATER**				
**Unit procedure**	**Unit cost (USD/MT)**	**Amount (MT/year)**	**Cost (USD/year)**	**%**
ST-101, Sterilization of BSG	**0.10**	861	86.06	100.00
**CHILLED WATER**				
**Unit procedure**	**Unit cost (USD/MT)**	**Amount (MT/year)**	**Cost (USD/year)**	**%**
BGBX-101, Machine for Shaping Snacks	0.50	1,941	970.54	100.00
**FREON**				
**Unit procedure**	**Unit cost (USD/MT)**	**Amount (MT/year)**	**Cost (USD/year)**	**%**
V-102, Tank for storage at 4°C of potatoes during 48 hours	0.15	68	10.26	100.00
**NATURAL GAS**				
**Unit procedure**	**Unit cost (USD/ MW-h)**	**Amount (MW-h /year)**	**Cost (USD/year)**	**%**
BGBX-101, Oven for the baking snacks	25.00	688	17,199.64	100.00

Table S8. Mass balance of the Process flow diagram (Figure 3)

**Stream**	**1**	**2**	**3**	**4**	**5**	**6**	**7**	**8**	**9**	**10**
**Type**	**Raw Material**									
**Total Mass Flow (Kg)**	1000	980	980	980	980	980	980	233.926	233.926	233.926
**BSG**	0	0	0	0	0	0	0	0	0	0
**BSG flour**	0	0	0	0	0	0	0	0	0	0
**Corn flour**	0	0	0	0	0	0	0	0	0	0
**Dough**	0	0	0	0	0	0	0	0	0	0
**impurities**	20	0	0	0	0	0	0	0	0	0
**moisture**	0	0	0	0	0	0	0	0	0	0
**N2**	0	0	0	0	0	0	0	0	0	0
**NaClO(aq)**	0	0	0	0	0	0	0	0	0	0
**O2**	0	0	0	0	0	0	0	0	0	0
**Potato disks**	0	0	980	980	980	980	980	233.926	233.926	0
**Potato flour**	0	0	0	0	0	0	0	0	0	233.926
**Residues**	0	0	0	0	0	0	0	0	0	0
**Snack**	0	0	0	0	0	0	0	0	0	0
**Sweet potato**	980	980	0	0	0	0	0	0	0	0
**Water**	0	0	0	0	0	0	0	0	0	0
**Wheat flour**	0	0	0	0	0	0	0	0	0	0

Table S8. Continued…

**Stream**	**11**	**12**	**13**	**14**	**15**	**16**	**17**	**18**	**19**	**20**
**Type**		**Raw Material**			**Emission**			**Solid Waste**		
**Total Mass Flow (Kg)**	233.926	230	230	230	174.9986	55.0014	55.0014	4.4216	50.5798	50.5798
**BSG**	0	51.612	51.612	51.612	0	51.612	0	0	0	0
**BSG flour**	0	0	0	0	0	0	51.612	1.0322	50.5798	50.5798
**Corn flour**	0	0	0	0	0	0	0	0	0	0
**Dough**	0	0	0	0	0	0	0	0	0	0
**impurities**	0	0	0	0	0	0	0	0	0	0
**moisture**	0	0	0	0	0	0	0	0	0	0
**N2**	0	0	0	0	0	0	0	0	0	0
**NaClO(aq)**	0	0	0	0	0	0	0	0	0	0
**O2**	0	0	0	0	0	0	0	0	0	0
**Potato disks**	0	0	0	0	0	0	0	0	0	0
**Potato flour**	233.926	0	0	0	0	0	0	0	0	0
**Residues**	0	0	0	0	0	0	0	0	0	0

**Snack**	0	0	0	0	0	0	0	0	0	0
**Sweet potato**	980	980	0	0	0	0	0	0	0	0
**Water**	0	0	0	0	0	0	0	0	0	0
**wheat flour**	0	0	0	0	0	0	0	0	0	0

Table S8. Continued...

**Stream**	**21**	**22**	**23**	**24**	**25**	**26**	**27**	**28**	**29**	**30**
**Type**	**Raw Material**		**Raw Material**		**Raw Material**			**Solid Waste**		**Emission**
**Total Mass Flow (Kg)**	342.74	342.74	250.54	250.54	878.89	0	1756.6758	17.5663	1739.1093	754.0072
**BSG**	0	0	0	0	0	0	0	0	0	0
**BSG flour**	0	0	0	0	0	0	50.5798	0	0.0024	0
**Corn flour**	342.74	342.74	0	0	0	0	342.74	0	0.0156	0
**Dough**	0	0	0	0	0	0	0	0	974.935	0
**impurities**	0	0	0	0	0	0	0	0	0	0
**moisture**	0	0	0	0	0	0	0	0	764.1382	751.7095
**N2**	0	0	0	0	0	0	0	0	0	1.7626
**NaClO(aq)**	0	0	0	0	0	0	0	0	0	0
**O2**	0	0	0	0	0	0	0	0	0	0.5351
**Potato disks**	0	0	0	0	0	0	0	0	0	0
**Potato flour**	0	0	0	0	0	0	233.926	0	0.0166	0
**Residues**	0	0	0	0	0	0	0	17.5663	0	0
**Snack**	0	0	0	0	0	0	0	0	0	0
**Sweet potato**	0	0	0	0	0	0	0	0	0	0
**Water**	0	0	0	0	878.89	0	878.89	0	0	0
**wheat flour**	0	0	250.54	250.54	0	0	250.54	0	0.0014	0

Table S8. Continued...

**Stream**	**31**	**S-103**	**A-1**	**A-2**	**V-1**	**V-2**	**W-1**	**R-1**	**R-2**
**Type**	**Revenue**	**Emission**	**Raw Material**	**Raw Material**	**Emission**	**Emission**			
**Total Mass Flow (Kg)**	986.3132	746.074	49.7353	0.0745	12.9175	0	69.7353	4993.5301	4923.7948
**BSG**	0	0	0	0	0	0	0	0	0
**BSG flour**	0.0024	0	0	0	0	0	0	0	0
**Corn flour**	0.0156	0	0	0	0	0	0	0	0
**Dough**	0	0	0	0	0	0	0	0	0
**impurities**	0	0	0	0	0	0	20	20	0
**moisture**	0	0	0	0	0	0	0	0	0
**N2**	0	0	0	0	9.8521	0	0	0	0

**NaClO(aq)**	0	0	0.0005	0	0	0	0.0005	0.0497	0.0492
**O2**	0	0	0	0	2.9909	0	0	0	0
**Potato disks**	0	0	0	0	0	0	0	0	0
**Potato flour**	0.0166	0	0	0	0	0	0	0	0
**Residues**	0	0	0	0	0	0	0	0	0
**Snack**	986.2772	0	0	0	0	0	0	0	0
**Sweet potato**	0	0	0	0	0	0	0	0	0
**Water**	0	746.074	49.7348	0.0745	0.0745	0	49.7348	4973.4804	4923.7456
**wheat flour**	0.0014	0	0	0	0	0	0	0	0

Table S8. Continued...

**Stream**	**R-3**	**R-4**	**R-5**
**Type**			
**Total Mass Flow (Kg)**	4973.5301	9999.9255	10000
**BSG**	0	0	0
**BSG flour**	0	0	0
**Corn flour**	0	0	0
**Dough**	0	0	0
**impurities**	0	0	0
**moisture**	0	0	0
**N2**	0	0	0
**NaClO(aq)**	0.0497	0	0
**O2**	0	0	0
**Potato disks**	0	0	0
**Potato flour**	0	0	0
**Residues**	0	0	0
**Snack**	0	0	0
**Sweet potato**	0	0	0
**Water**	4973.4804	9999.9255	10000
**wheat flour**	0	0	0
