# Supplementary material for: Techno-Economic Feasibility of Functional Snacks from Brewer’s Spent Grain and Sweet Potato: A Simulation Study
Source: Foods. 2026 May 9;15(10):1654. doi: 10.3390/foods15101654 (PMC13205998; doi:10.3390/foods15101654)
Supplement: Supplementary file 1 [file foods-15-01654-s001.zip › Supplementary Material File S2-TEA.pdf]

Supplementary Material: Extended Discussion on Regulatory Aspects and Product-Related Considerations

This Supplementary Material provides extended background information on regulatory aspects, safety considerations, and product-related constraints associated with the proposed snack formulation. These discussions were part of the initial version of the manuscript and have been relocated to this section to maintain a clear focus in the main text on process simulation, techno-economic analysis, and industrial feasibility. The information presented here complements the main findings by providing additional context on factors relevant to product development and commercialization.

This supplementary section should be read in conjunction with Sections 3.2 and 3.3 of the main manuscript.

S.1 Supplementary section to point 3.2 Regulatory and Process Safety Considerations

Although the proposed snack is economically viable, several considerations should be considered. In many jurisdictions, BSG is classified as a novel food and requires pre-market authorization under the applicable regulatory framework. Furthermore, the finished product must comply with the labeling standards of the destination market (e.g., Regulation (EU) 1169/2011 or applicable FDA requirements). Such standards typically mandate the declaration of allergens, given that although BSG contains low levels of gluten, its botanical origin from barley may necessitate explicit disclosure, complete nutritional labeling, and specification of the country of origin. Additionally, some of the final protein content in BSG will consist of allergens and antinutrients, such as hordeins, serine protease inhibitors, trypsin, and alpha-amylase inhibitors, as well as the allergen glyceroldehyde-3-phosphate dehydrogenase 1 [1] .

The safety and quality of the snack depend on the absence of toxic chemicals and contaminants, as well as on the quality of the raw materials. For example, the presence of heavy metals, such as

cadmium, lead, and pesticide residues, which were concentrated during the brewing process and BSG dehydration. Additionally, mycotoxins such as deoxynivalenol, zearalenone, and type B trichothecenes produced by *Fusarium* [2] will be considered and included in a Hazard Analysis and Critical Control Points plan, in compliance with regulations. The quality issues mentioned above also apply to the other flours used in preparing the snack, such as wheat and corn.

From a microbiological perspective, BSG stabilization and dehydration are essential for reducing water activity and preventing the growth of pathogenic and spoilage microbes, including *Salmonella* spp., *Listeria monocytogenes*, *Escherichia coli,* and *Staphylococcus aureus*. Also, the dehydration process involves some aspects in the final nutritional and sensorial quality [3].

S2. Supplementary section to point 3.3 Process Limitations, Product Constraints, and Future Work

Within this evolving market context, the use of BSG in food products poses several technological challenges, particularly regarding texture, color, and shelf life. These challenges stem primarily from BSG's high dietary fiber and protein content. One of the most recurrent issues is the marked increase in product hardness and structural compactness [4]. Because BSG contains significant amounts of insoluble fiber and proteins that rapidly absorb water, it competes with starch and other ingredients for available moisture. This competition results in denser, more rigid matrices rather than the soft, aerated structures typically associated with starch-based systems [5]. For example, in baked goods, a 17% inclusion of BSG has been shown to significantly harden cookies and create more compact structures [4]. In breads, BSG can weaken or dilute the gluten network, producing firmer crumbs and, in some cases, accelerating staling during storage. To mitigate these textural drawbacks, researchers have explored pretreatments, including fermentation and enzymatic hydrolysis.

Fermented or enzyme-modified BSG has demonstrated meaningful improvements, including reduced hardness and softer crumbs in baked applications; in muffins, BSG protein hydrolysates have been especially effective in enhancing softness [6].

Color is another key challenge when incorporating BSG. Due to its naturally dark brown hue, BSG contributes to visibly darker finished products, a change that becomes more pronounced at higher substitution levels [7]. This darkening is accompanied by shifts in chromatic parameters, often increasing the a* value toward red and decreasing the b* value toward yellow. Beyond its intrinsic pigmentation, BSG promotes Maillard reactions during high-temperature processing, as its proteins and reducing sugars interact to form brown-colored melanoidins [8]. Although these reactions enhance flavor complexity, they may negatively influence consumer acceptance if the final product appears overly dark.

Despite these sensory and structural complications, BSG provides notable advantages for shelf-life extension. Its fiber- and protein-rich matrix tends to reduce moisture content and water activity in various products, including cookies and cereal bars. For instance, in cookies, increasing BSG levels has been associated with moisture reductions to approximately 3.37% and Aw values around 0.506. Such decreases are critical for limiting microbial growth and significantly enhancing microbiological stability [5,8]. Furthermore, the protein matrix in BSG can serve as a protective barrier against microbial degradation of starch. At the same time, the high fiber content offers a substrate that microorganisms cannot efficiently metabolize. Although the effect on texture stability during storage can vary—higher BSG levels may intensify crumb firming—strategies such as using fermented BSG (e.g., sourdough-based systems) have proven effective in delaying staling [9].

Consumers' sensory acceptance of foods formulated with BSG largely depends on the inclusion level and its impact on key attributes such as texture, color, and flavor. Overall, consumer studies consistently indicate that products formulated with low to moderate BSG levels achieve good acceptance, whereas higher levels often lead to noticeable declines in liking [10,11]. This pattern underscores both the potential and the challenges of integrating BSG as an innovative, upcycled ingredient in food product development. Most research suggests that the optimal acceptance range is 15–20% flour substitution, which preserves sensory quality while delivering meaningful nutritional benefits. For example, cookies formulated with 0% and 8% extruded BSG show no significant differences in consumer liking.

In comparison, bread maintains an optimal balance between sensory performance and nutritional enhancement with up to 10% substitution [4]. Remarkably, cookies enriched with BSG from durum or soft wheat varieties have achieved the highest overall quality scores—particularly at 30% substitution—attributed to a stronger freshly baked flavor and reduced hardness and fibrousness. In muffins, formulations containing 2% BSG protein hydrolysate reach acceptance levels comparable to controls [6]. Similarly, cereal bars with intermediate BSG levels (e.g., 7.74% and 12.69%) receive high liking scores, suggesting that the sensory experience itself can outweigh concerns or preconceptions about using this unconventional ingredient.

Conversely, higher inclusion levels, typically 17% or more, often reduce acceptability due to increased hardness, darker appearance, and more noticeable flavor deviations. Consumers have reported that cookies with 17% BSG have off-flavors and an undesirable aftertaste, and fewer panelists rate them as "delicious" [4]. Together, these findings highlight not only BSG's

promise as a sustainable, forward-thinking ingredient but also the importance of carefully optimizing its incorporation to maintain desirable sensory properties. A preliminary sensory evaluation of this prototype snack—formulated with 6.25% BSG, wheat, sweet potato flour, and corn — was done in previous work (Gómez-Cisneros et al., 2025). In that study, the product showed favorable overall acceptance among participants, with no evident negative sensory attributes. Although sensory analysis was not the primary focus of this study, these observations suggest that the incorporation of BSG at the evaluated level did not adversely affect perceived quality. These findings highlight promising consumer interest and underscore the market potential for innovative snacks made with sustainable ingredients such as BSG combined with wheat, corn, and sweet potatoes.

Finally, consuming whole-grain snacks has multiple health-promoting benefits. Figure S1 depicts some positive metabolic outcomes when BSG is incorporated into various food matrices. Several combinations of grains with BSG can enhance these health benefits and promote sustainable food production by integrating circular-economy principles.

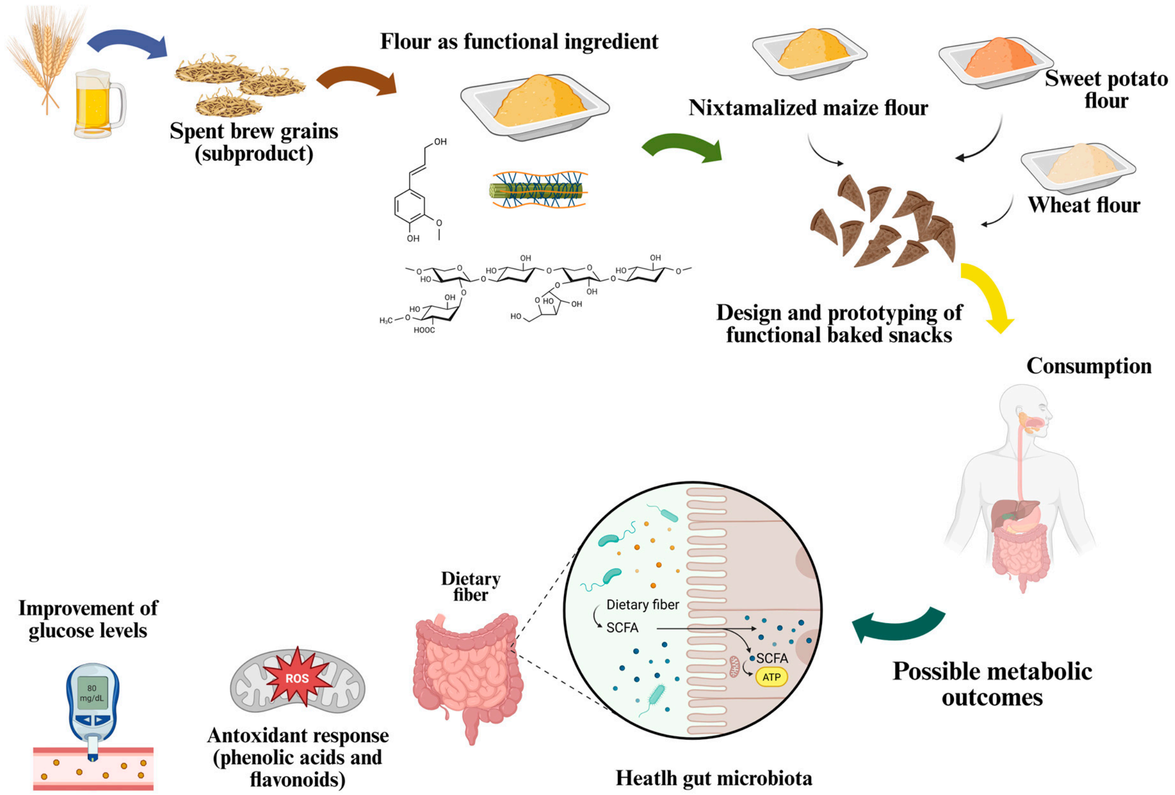

Figure S1. Use of beer production residues to produce functional ingredients for snacks with possible health benefits.

BSG is considered a good source of dietary fiber, including both insoluble and soluble fibers [12]. Specifically, cellulose, hemicellulose, lignin, and arabinoxylans constitute significant proportions of dietary fiber [13]. The fiber in BSG can promote gut health by modulating the gut microbiome composition, stimulating the growth of beneficial bacteria, and inhibiting pathogenic bacteria [14]. The high fiber content of BSG may also contribute to increased satiety and short-term appetite control and may aid in weight management [4].

It has been reported that BSG is a source of polyphenols, including flavonoids, which are recognized for their antioxidant properties [15]. Polyphenols from BSG can help protect against oxidative stress and reduce the risk of chronic diseases, including cardiovascular

disease, certain cancers, and neurodegenerative diseases [16]. Free and bound phenolic compounds have been reported in BSG, such as ferulic acid, p-coumaric acid, synaptic acid, caffeic acid, syringic acid, catechin, epicatechin, quinic acid, and gallic acid [17]. Nevertheless, the bioavailability and bioaccessibility of polyphenols from BSG can be influenced by the food matrix and interactions with other macronutrients [18].

Another significant effect of the use and production of foods with BSG is its reduction in glycemic response potential (due to their resistant starch and fiber content). In this sense, the high fiber and resistant starch content of BSG can contribute to a reduced glycemic response, which may be beneficial for individuals with metabolic health concerns, such as diabetes [19]. Incorporating BSG into food products can enhance their nutritional profile and potentially improve the dietary attributes of commonly consumed foods [20].

The available evidence suggests that dietary fiber, polyphenols, and other bioactive compounds in BSG, such as xylooligosaccharides, have prebiotic effects, the potential to promote gut health [13], antioxidant benefits, the ability to modulate glycemic response, and the potential to contribute to weight management and overall metabolic health. Further research is needed to elucidate the mechanisms underlying BSG's health-promoting effects fully and to optimize its use as a health-promoting food ingredient.

SUPLEMENTARY REFERENCES

1. Gregersen Echers, S.; Mikkelsen, R.K.; Abdul-Khalek, N.; Queiroz, L.S.; Hobley, T.J.; Schulz, B.L.; Overgaard, M.T.; Jacobsen, C.; Yesiltas, B. Residual Barley Proteins in Brewers' Spent Grains: Quantitative Composition and Implications for Food Ingredient

Applications. *Innovative Food Science & Emerging Technologies* **2025**, *106*, 104277, doi:https://doi.org/10.1016/j.ifset.2025.104277.

2. Pack, E.D.; Meyerhoff, K.; Schmale III, D.G. Tracking Zearalenone and Type-B Trichothecene Mycotoxins in the Commercial Production of Beer and Brewers' Spent Grains. *Journal of the American Society of Brewing Chemists* **2022**, *80*, 180–189, doi:10.1080/03610470.2021.1938489.
3. Carella, A.; Lamacchia, C. Drying Techniques for the Valorization of Brewer's Spent Grains: Impacts on Nutritional Quality, Sensory Properties, and Process Efficiency. A Review. *Applied Food Research* **2025**, *5*, 101429, doi:https://doi.org/10.1016/j.afres.2025.101429.
4. Gutierrez-Barrutia, M.B.; Cozzano, S.; Arcia, P.; del Castillo, M.D. An Insight into the Use of Extruded Brewers' Spent Grain as a Healthy Human Snack Ingredient. Effects on Food Structure, Sensory Quality, Satiety and Gastrointestinal Tolerance. *Food Biosci.* **2025**, *63*, 105583, doi:https://doi.org/10.1016/j.fbio.2024.105583.
5. Nicolai, M.; Palma, M.L.; Reis, R.; Amaro, R.; Fernandes, J.; Gonçalves, E.M.; Silva, M.; Lageiro, M.; Charmier, A.; Maurício, E.; et al. Assessing the Potential of Brewer's Spent Grain to Enhance Cookie Physicochemical and Nutritional Profiles. *Foods* **2025**, *14*, doi:10.3390/foods14010095.
6. Bazsefidpar, N.; Ghandehari Yazdi, A.P.; Karimi, A.; Yahyavi, M.; Amini, M.; Ahmadi Gavlighi, H.; Simal-Gandara, J. Brewers Spent Grain Protein Hydrolysate as a Functional Ingredient for Muffins: Antioxidant, Antidiabetic, and Sensory Evaluation. *Food Chem.* **2024**, *435*, 137565, doi:https://doi.org/10.1016/j.foodchem.2023.137565.
7. Paciulli, M.; Sogari, G.; Rodolfi, M.; Parenti, O.; Andreani, G.; Chiavaro, E. Fostering Circular Economy: Brewing By-Products as Innovative Ingredients for Cereal Bar Formulation. *Foods* **2024**, *13*, doi:10.3390/foods13152355.
8. Aradwad, P.; Raut, S.; Abdelfattah, A.; Rauh, C.; Sturm, B. Brewer's Spent Grain: Unveiling Innovative Applications in the Food and Packaging Industry. *Compr. Rev. Food Sci. Food Saf.* **2025**, *24*, e70150, doi:https://doi.org/10.1111/1541-4337.70150.
9. Pérez-Alva, A.; Martín-del-Campo, S.T.; Baigts-Allende, D.K. Brewer's Spent Grain (BSG) as an Ingredient for Leavened Bread Making: Challenges and Opportunities. *J. Cereal Sci.* **2025**, *124*, 104223, doi:https://doi.org/10.1016/j.jcs.2025.104223.
10. Virdi, A.S.; Mahajan, A.; Devraj, M.; Sanghi, R. Brewers' Spent Grains: Techno-Functional Challenges and Opportunity in the Valorization for Food Products. *LWT* **2025**, *227*, 117785, doi:https://doi.org/10.1016/j.lwt.2025.117785.
11. Saberian, H.; Ghandehari Yazdi, A.P.; Nejatian, M.; Bazsefidpar, N.; Mohammadian, A.H.; Rahmati, M.; Assadpour, E.; Jafari, S.M. Brewers' Spent Grain as a Functional Ingredient in Bakery, Pasta, and Cereal-Based Products. *Future Foods* **2024**, *10*, 100479, doi:https://doi.org/10.1016/j.fufo.2024.100479.

12. Gmoser, R.; Fristedt, R.; Larsson, K.; Undeland, I.; Taherzadeh, M.J.; Lennartsson, P.R. From Stale Bread and Brewers Spent Grain to a New Food Source Using Edible Filamentous Fungi. *Bioengineered* **2020**, *11*, 582–598, doi:10.1080/21655979.2020.1768694.

13. Chinbat, O.; Erdenetsog, P.; Tuvshintur, B.; Gantumur, A.; Burenjargal, M.; Chimeddorj, B.; Janlav, M. In Vitro and in Vivo Investigation of the Biological Action of Xylooligosaccharides Derived from Industrial Waste. *Food Sci. Nutr.* **2024**, *12*, 7877–7884, doi:https://doi.org/10.1002/fsn3.4391.

14. Ozdal, T.; Sela, D.A.; Xiao, J.; Boyacioglu, D.; Chen, F.; Capanoglu, E. The Reciprocal Interactions between Polyphenols and Gut Microbiota and Effects on Bioaccessibility. *Nutrients* **2016**, *8*, doi:10.3390/nu8020078.

15. Nyhan, L.; Sahin, A.W.; Schmitz, H.H.; Siegel, J.B.; Arendt, E.K. Brewers' Spent Grain: An Unprecedented Opportunity to Develop Sustainable Plant-Based Nutrition Ingredients Addressing Global Malnutrition Challenges. *J. Agric. Food Chem.* **2023**, *71*, 10543–10564, doi:10.1021/acs.jafc.3c02489.

16. Birsan, R.I.; Wilde, P.; Waldron, K.W.; Rai, D.K. Recovery of Polyphenols from Brewer's Spent Grains. *Antioxidants* **2019**, *8*, doi:10.3390/antiox8090380.

17. Ikram, S.; Huang, L.; Zhang, H.; Wang, J.; Yin, M. Composition and Nutrient Value Proposition of Brewers Spent Grain. *J. Food Sci.* **2017**, *82*, 2232–2242, doi:https://doi.org/10.1111/1750-3841.13794.

18. Diez-Sánchez, E.; Quiles, A.; Hernando, I. Interactions between Blackcurrant Polyphenols and Food Macronutrients in Model Systems: In Vitro Digestion Studies. *Foods* **2021**, *10*, doi:10.3390/foods10040847.

19. Ullah, H.; Esposito, C.; Piccinocchi, R.; De Lellis, L.F.; Santarcangelo, C.; Minno, A. Di; Baldi, A.; Buccato, D.G.; Khan, A.; Piccinocchi, G.; et al. Postprandial Glycemic and Insulinemic Response by a Brewer's Spent Grain Extract-Based Food Supplement in Subjects with Slightly Impaired Glucose Tolerance: A Monocentric, Randomized, Cross-Over, Double-Blind, Placebo-Controlled Clinical Trial. *Nutrients* **2022**, *14*, doi:10.3390/nu14193916.

20. Naibaho, J.; Korzeniowska, M. Brewers' Spent Grain in Food Systems: Processing and Final Products Quality as a Function of Fiber Modification Treatment. *J. Food Sci.* **2021**, *86*, 1532–1551, doi:https://doi.org/10.1111/1750-3841.15714.
